# Supplementary material for: Acidic pH reduces agonist efficacy and responses to synaptic-like glycine applications in zebrafish α1 and rat α1β recombinant glycine receptors
Source: J Physiol. Author manuscript; Available in PMC 2022 Feb 16. (PMC8836455; doi:10.1113/JP282171)
Supplement: SuppMat [file EMS142060-supplement-SuppMat.docx]

**Manuscript Title:** Acidic pH reduces agonist efficacy and responses to synaptic-like glycine applications in zebrafish α1 and rat α1β recombinant glycine receptors

**Authors:** Josip Ivica, Remigijus Lape and Lucia Sivilotti

**Animal model used, if applicable:** not applicable

**Underlying hypothesis:** This investigation tests the hypothesis that acidic extracellular pH affects glycine receptor responses to agonists in whole cell, outside out and cell-attached recordings. Different aspects of the responses are considered (see individual questions)

**Definitions of ‘n’:**

Question 1: n = number of individual concentration-response curves, each obtained from a different cell.

Question 2: n = number of single-channel activity clusters recorded.

Question 3: n = number of outside-out patches.

**Statistical summary table:**

| Experimental question number* | Finding/ conclusion | Experimental location/ variable  e.g. muscle, neocortex or genotype | Mean value  (or other summary statistic) | SD | n val. | P** | Units | Data comparisons  e.g. WT vs KO | Statistical test | Any other variable  e.g. subjects’ age or sex | Figure/ table in which data are presented | Comments  e.g. observation |
| --- | --- | --- | --- | --- | --- | --- | --- | --- | --- | --- | --- | --- |
| Question 1: Concentration-response curve *EC*_50_? | Acidic pH increases EC_50_ of GlyR  α1 | Glycine zf α1 GlyR pH 6.4 | 680 | 110 | 7 | <10-^6^ | µM | vs Glycine zf α1 GlyR pH 7.4 | Unpaired *t*-test |  | Figure1/  Table1 | Hill equation fits to agonist concentration-response data from whole-cell recordings; holding potential −40 mV, internal chloride 30 mM. |
|  |  | Glycine zf α1 GlyR  pH 7.4 | 190 | 60 | 8 | / | µM | / |  |  |  |  |
|  |  | Glycine zf α1 GlyR  pH 8.4 | 280 | 110 | 7 | 0.087 | µM | vs Glycine zf α1 GlyR pH 7.4 | Unpaired *t*-test |  |  |  |
|  |  | β-alanine zf α1 GlyR  pH 6.4 | 1890 | 750 | 9 | 0.00002 | µM | vs β-alanine zf α1 GlyR  pH 7.4 | Unpaired *t*-test |  |  |  |
|  |  | β-alanine zf α1 GlyR  pH 7.4 | 340 | 190 | 10 | / | µM | / |  |  |  |  |
|  |  | β-alanine zf α1 GlyR  pH 8.4 | 990 | 260 | 6 | 0.0001 | µM | vs β-alanine zf WT α1  pH 7.4 | Unpaired *t*-test |  |  |  |
|  |  | Taurine zf α1 GlyR  pH 6.4 | 3200 | 700 | 5 | 0.0003 | µM | vs Taurine zf α1 GlyR  pH 7.4 | Unpaired *t*-test |  |  |  |
|  |  | Taurine zf α1 GlyR  pH 7.4 | 1050 | 220 | 6 | / | µM | / |  |  |  |  |
|  |  | Taurine zf α1 GlyR  pH 8.4 | 3400 | 1400 | 5 | 0.0027 | µM | vs Taurine zf α1 GlyR  pH 7.4 | Unpaired *t*-test |  |  |  |
| Question 2.  Does extracellular pH affects single channel maximum open probability? | Extracellular pH 6.4 reduces maximal open probability in homomeric zf a1 GlyR | Glycine zf α1 GlyR ; pH 6.4 | 0.93 | 0.11 | 30 | *P*=0.0012 |  | vs Glycine zf α1 GlyR ; pH 7.4 | randomisation test |  | Figure 3/  Table 2 | Cell-attached single channel recordings; Vcomm= +100 mV; open/shut transitions detected using half-amplitude threshold algorithm. |
|  |  | Glycine zf α1 GlyR ; pH 7.4 | 0.97 | 0.05 | 48 | / |  | / |  |  | Figure 3/  Table 2 |  |
|  |  | Glycine zf a1 GlyR ; pH 8.4 | 0.96 | 0.05 | 35 | 0.1836 |  | Vs Glycine zf α1 GlyR ; pH 7.4 |  |  | Figure 3/  Table 2 |  |
|  |  | β-alanine zf α1 GlyR ; pH 6.4 | 0.57 | 0.24 | 55 | < 10^-6^ |  | vs β-alanine zf α1 GlyR ; pH 7.4 |  |  | Figure 3/  Table 2 |  |
|  |  | β-alanine zf α1 GlyR ; pH 7.4 | 0.91 | 0.21 | 30 | / |  | / |  |  | Figure 3 /  Table 2 |  |
|  |  | β-alanine zf α1 GlyR ; pH 8.4 | 0.81 | 0.18 | 43 | 0.047 |  | vs β-alanine zf α1 GlyR ; pH 7.4 |  |  | Figure 3 /  Table 2 |  |
|  |  | Taurine zf α1 GlyR ; pH 6.4 | 0.34 | 0.20 | 49 | <<10^-6^ |  | vs Taurine zf α1 GlyR ; pH 7.4 |  |  | Figure 3 /  Table 2 |  |
|  |  | Taurine zf α1 GlyR ; pH 7.4 | 0.66 | 0.24 | 71 | / |  | / |  |  | Figure 3 /  Table 2 |  |
|  |  | Taurine zf α1 GlyR ; pH 8.4 | 0.62 | 0.23 | 47 | 0.391 |  | vs. Taurine zf α1 GlyR ; pH 7.4 |  |  |  |  |
| Question 3  Is conductance of zf GlyR α1 affected at pH 6.4? | Conductance of GlyR at pH 6.4 is not different from conductance at pH 7.4 | zf α1 GlyR; conductance at pH 7.4 | 73.8 | 6.4 | 4 | / | pS | / | Unpaired *t*-test |  | Figure 3 | Outside out recordings of zebrafish a1 GlyR in the presence of 10 mM glycine under 131.1 mM intracellular chloride |
|  |  | zf α1 GlyR; conductance at pH 6.4 | 71.9 | 2.9 | 4 | 0.6114 | pS | vs zf α1 GlyR; conductance at pH 7.4 |  |  |  |  |
| Question 4  Is the amplitude of outside-out responses to agonist concentration jumps affected by pH 6.4? | Reduced amplitude in acidic pH 6.4 both in homomeric and heteromeric GlyR.  The effect is consistent across all agonists and internal chloride concentrations | Glycine zf α1 GlyR;pH 7.4 | 1160 | 630 | 6 | / | pA | / | Paired *t*-test | 131.1 mM intracellular chloride | Figure 4/  Table 3 | 2 ms pulses of agonists glycine-alanine, taurine and GABA ( 3 -100mM) to outside-out patches using three intracellular chloride solutions (131.1, 30 and 10 mM). |
|  |  | Glycine zf α1 GlyR;pH 6.4 | 970 | 520 | 6 | 0.016 | pA | vs Glycine zf α1 GlyR;pH 7.4 |  | 131.1 mM intracellular chloride | Figure 4/  Table 3 |  |
|  |  | Glycine rat α1/β GlyR;pH 7.4 | 348 | 200 | 7 | / | pA | / |  | 131.1 mM intracellular chloride | Table 4 |  |
|  |  | Glycine rat α1/β GlyR;pH 6.4 | 318 | 200 | 7 | 0.013 | pA | vs Glycine rat α1/β GlyR;pH 7.4 |  | 131.1 mM intracellular chloride | Table 4 |  |
|  |  | β-alanine zf α1 GlyR;pH 7.4 | 910 | 280 | 6 | / | pA | / |  | 131.1 mM intracellular chloride | Figure 4/  Table 3 |  |
|  |  | β-alanine zf α1 GlyR;pH 6.4 | 630 | 210 | 6 | 0.019 | pA | vs.β-alanine zf α1 GlyR;pH 7.4 |  | 131.1 mM intracellular chloride | Figure 4/  Table 3 |  |
|  |  | Taurine zf α1 GlyR;pH 7.4 | 970 | 225 | 6 | / | pA | / |  | 131.1 mM intracellular chloride | Figure 4/  Table 3 |  |
|  |  | Taurine zf α1 GlyR;pH 6.4 | 570 | 180 | 6 | 0.010 | pA | vs Taurine zf α1 GlyR;pH 7.4 |  | 131.1 mM intracellular chloride | Figure 4/  Table 3 |  |
|  |  | GABA zf α1 GlyR;pH 7.4 | 1170 | 430 | 5 | / | pA | / |  | 131.1 mM intracellular chloride | Figure 4/  Table 3 |  |
|  |  | GABA zf α1 GlyR;pH 7.4 | 480 | 97 | 5 | 0.014 | pA | vs GABA zf α1 GlyR;pH 7.4 |  | 131.1 mM intracellular chloride | Figure 4/  Table 3 |  |
|  |  | Glycine zf α1 GlyR;pH 7.4 | 746 | 620 | 11 | / | pA | / |  | 30 mM intracellular chloride | Figure 6/  Table 4 |  |
|  |  | Glycine zf α1 GlyR;pH 6.4 | 498 | 540 | 11 | 0.002 | pA | vs Glycine zf α1 GlyR;pH 7.4 |  | 30 mM intracellular chloride | Figure 6/  Table 4 |  |
|  |  | Taurine zf α1 GlyR;pH 7.4 | 354 | 183 | 7 | / | pA | / |  | 30 mM intracellular chloride | Figure 6 |  |
|  |  | Taurine zf α1 GlyR;pH 6.4 | 112 | 55 | 7 | 0.003 | pA | vs Taurine zf α1 GlyR;pH 7.4 |  | 30 mM intracellular chloride | Figure 6 |  |
|  |  | Glycine zf α1 GlyR;pH 7.4 | 312 | 375 | 7 | / | pA | / |  | 10 mM intracellular chloride | Figure 7/  Table 4 |  |
|  |  | Glycine zf α1 GlyR;pH 6.4 | 199 | 259 | 7 | 0.046 | pA | vs Glycine zf α1 GlyR;pH 7.4 |  | 10 mM intracellular chloride | Figure 7/  Table 4 |  |
|  |  | Glycine rat α1/β GlyR;pH 7.4 | 106 | 48 | 7 | / | pA | / |  | 10 mM intracellular chloride | Figure 7/  Table 4 |  |
|  |  | Glycine rat α1/β GlyR;pH 6.4 | 79 | 35 | 7 | 0.003 | pA | vs Glycine rat α1/β GlyR;pH 7.4 |  | 10 mM intracellular chloride | Figure 7/  Table 4 |  |
| Question 5  Is the decay of outside-out responses to agonist concentration jumps affected by pH 6.4? | Decay time constant becomes shorter in acidic pH both for homomeric and heteromeric GlyRs | Glycine zf α1 GlyR;pH 7.4 | 25.5 | 8.1 | 6 | / | ms | / | Paired *t*-test | 131.1 mM intracellular chloride | Figure 4/  Table 3 |  |
|  |  | Glycine zf α1 GlyR;pH 6.4 | 9.7 | 4.3 | 6 | 0.012 | ms | vs Glycine zf α1 GlyR;pH 7.4 |  | 131.1 mM intracellular chloride | Figure 4/  Table 3 |  |
|  |  | Glycine rat α1/β GlyR;pH 7.4 | 16.5 | 8.8 | 7 | / | ms | / |  | 131.1 mM intracellular chloride | Table 4 |  |
|  |  | Glycine rat α1/β GlyR;pH 6.4 | 10.5 | 8.5 | 7 | 0.001 | ms | vs Glycine rat α1/β GlyR;pH 7.4 |  | 131.1 mM intracellular chloride | Table 4 |  |
|  |  | β-alanine zf α1 GlyR;pH 7.4 | 5.7 | 2.0 | 6 | / | ms | / |  | 131.1 mM intracellular chloride | Figure 4/  Table 3 |  |
|  |  | β-alanine zf α1 GlyR;pH 6.4 | 2.6 | 0.8 | 6 | 0.002 | ms | vs β-alanine zf α1 GlyR;pH 7.4 |  | 131.1 mM intracellular chloride | Figure 4/  Table 3 |  |
|  |  | Taurine zf α1 GlyR;pH 7.4 | 4.3 | 1.1 | 6 | / | ms | / |  | 131.1 mM intracellular chloride | Figure 4/  Table 3 |  |
|  |  | Taurine zf α1 GlyR;pH 6.4 | 2.3 | 0.7 | 6 | 0.006 | ms | vs Taurine zf α1 GlyR;pH 7.4 |  | 131.1 mM intracellular chloride | Figure 4/  Table 3 |  |
|  |  | GABA zf α1 GlyR;pH 7.4 | 2.5 | 0.5 | 5 | / | ms | / |  | 131.1 mM intracellular chloride | Figure 4/  Table 3 |  |
|  |  | GABA zf α1 GlyR;pH 7.4 | 1.3 | 0.3 | 5 | 0.002 | ms | vs GABA zf α1 GlyR;pH 7.4 |  | 131.1 mM intracellular chloride | Table 3/ Figure4 |  |
|  |  | Glycine zf α1 GlyR;pH 7.4 | 17.8 | 10.6 | 11 | / | ms | / |  | 30 mM intracellular chloride | Figure 6/  Table 4 |  |
|  |  | Glycine zf α1 GlyR;pH 6.4 | 8.1 | 3.9 | 11 | 0.004 | ms | vs Glycine zf α1 GlyR;pH 7.4 |  | 30 mM intracellular chloride | Figure 6/  Table 4 |  |
|  |  | Taurine zf α1 GlyR;pH 7.4 | 2.5 | 0.8 | 7 | / | ms | / |  | 30 mM intracellular chloride | Figure 6 |  |
|  |  | Taurine zf α1 GlyR;pH 6.4 | 1.5 | 0.6 | 7 | 0.002 | ms | vs Taurine zf α1 GlyR;pH 7.4 |  | 30 mM intracellular chloride | Figure 6 |  |
|  |  | Glycine rat α1/β GlyR;pH 7.4 | 10.4 | 3.7 | 8 | / | ms | / |  | 30 mM intracellular chloride | Figure 7  Table 4 |  |
|  |  | Glycine rat α1/β GlyR;pH 6.4 | 6.4 | 1.5 | 8 | 0.010 | ms | vs Glycine rat α1/β GlyR;pH 7.4 |  | 30 mM intracellular chloride | Figure 7  Table 4 |  |
|  |  | Glycine rat α1/β GlyR;pH 7.4 | 7.6 | 2.2 | 7 | / | ms | / |  | 10 mM intracellular chloride | Figure 7/  Table 4 |  |
|  |  | Glycine rat α1/β GlyR;pH 6.4 | 4.5 | 1.4 | 7 | 0.013 | ms | vs Glycine rat α1/β GlyR;pH 7.4 |  | 10 mM intracellular chloride | Figure 7/  Table 4 |  |
|  |  | Glycine zf α1 GlyR;pH 7.4 | 10.6 | 2.5 | 7 | / | ms | / |  | 10 mM intracellular chloride | Figure 7/  Table 4 |  |
|  |  | Glycine zf α1 GlyR;pH 6.4 | 4.5 | 2.4 | 7 | 0.0001 | ms | vs Glycine zf α1 GlyR;pH 7.4 |  | 10 mM intracellular chloride | Figure 7/  Table 4 |  |
| Question 6  Is the rise time of outside-out responses to agonist concentration jumps affected by pH 6.4? | Rise time constants become slower in acidic pH both for homomeric and heteromeric GlyRs | Glycine zf α1 GlyR;pH 7.4 | 0.17 | 0.07 | 6 | / | ms | / | Paired *t*-test | 131.1 mM intracellular chloride | Figure 4/  Table3 |  |
|  |  | Glycine zf α1 GlyR;pH 6.4 | 0.29 | 0.12 | 6 | 0.037 | ms | vs Glycine zf α1 GlyR;pH 7.4 |  | 131.1 mM intracellular chloride | Figure 4/  Table3 |  |
|  |  | Glycine rat α1/β GlyR;pH 7.4 | 0.20 | 0.06 | 7 | / | ms | / |  | 131.1 mM intracellular chloride | Table4 |  |
|  |  | Glycine rat α1/β GlyR;pH 6.4 | 0.27 | 0.08 | 7 | 0.001 | ms | vs Glycine rat α1/β GlyR;pH 7.4 |  | 131.1 mM intracellular chloride | Table 4 |  |
|  |  | β-alanine zf α1 GlyR;pH 7.4 | 0.19 | 0.04 | 6 | / | ms | / |  | 131.1 mM intracellular chloride | Figure 4/  Table 3 |  |
|  |  | β-alanine zf α1 GlyR;pH 6.4 | 0.35 | 0.10 | 6 | 0.003 | ms | vs β-alanine zf α1 GlyR;pH 7.4 |  | 131.1 mM intracellular chloride | Figure 4/  Table 3 |  |
|  |  | Taurine zf α1 GlyR;pH 7.4 | 0.23 | 0.05 | 6 | / | ms | / |  | 131.1 mM intracellular chloride | Figure 4/  Table 3 |  |
|  |  | Taurine zf α1 GlyR;pH 6.4 | 0.40 | 0.10 | 6 | 0.003 | ms | vs Taurine zf α1 GlyR;pH 7.4 |  | 131.1 mM intracellular chloride | Figure 4/  Table 3 |  |
|  |  | GABA zf α1 GlyR;pH 7.4 | 0.35 | 0.06 | 5 | / | ms | / |  | 131.1 mM intracellular chloride | Figure 4/  Table 3 |  |
|  |  | GABA zf α1 GlyR;pH 7.4 | 0.57 | 0.18 | 5 | 0.02 | ms | vs GABA zf α1 GlyR;pH 7.4 |  | 131.1 mM intracellular chloride | Figure 4/  Table 3 |  |
|  |  | Glycine zf α1 GlyR;pH 7.4 | 0.24 | 0.06 | 11 | / | ms | / |  | 30 mM intracellular chloride | Figure 6/  Table 4 |  |
|  |  | Glycine zf α1 GlyR;pH 6.4 | 0.44 | 0.18 | 11 | 0.001 | ms | vs Glycine zf α1 GlyR;pH 7.4 |  | 30 mM intracellular chloride | Figure 6/  Table 4 |  |
|  |  | Taurine zf α1 GlyR;pH 7.4 | 0.24 | 0.07 | 7 | / | ms | / |  | 30 mM intracellular chloride | Figure 6 |  |
|  |  | Taurine zf α1 GlyR;pH 6.4 | 0.44 | 0.11 | 7 | 0.0003 | ms | vs Taurine zf α1 GlyR;pH 7.4 |  | 30 mM intracellular chloride | Figure 6 |  |
|  |  | Glycine rat α1/β GlyR;pH 7.4 | 0.27 | 0.13 | 8 | / | ms | / |  | 30 mM intracellular chloride | Figure 7/  Table 4 |  |
|  |  | Glycine rat α1/β GlyR;pH 6.4 | 0.37 | 0.18 | 8 | 0.004 | ms | vs Glycine rat α1/β GlyR;pH 7.4 |  | 30 mM intracellular chloride | Figure 7/  Table 4 |  |
|  |  | Glycine rat α1/β GlyR;pH 7.4 | 0.22 | 0.06 | 7 | / | ms | / |  | 10 mM intracellular chloride | Figure 7/  Table 4 |  |
|  |  | Glycine rat α1/β GlyR;pH 6.4 | 0.32 | 0.08 | 7 | 0.002 | ms | vs Glycine rat α1/β GlyR;pH 7.4 |  | 10 mM intracellular chloride | Figure 7/  Table 4 |  |
|  |  | Glycine zf α1 GlyR;pH 7.4 | 0.26 | 0.09 | 7 | / | ms | / |  | 10 mM intracellular chloride | Figure 7/  Table 4 |  |
|  |  | Glycine zf α1 GlyR;pH 6.4 | 0.38 | 0.18 | 7 | 0.186 | ms | vs Glycine zf α1 GlyR;pH 7.4 |  | 10 mM intracellular chloride | Figure 7/  Table 4 |  |

*You may use multiple lines for the same question to indicate multiple comparisons

** Authors may wish to make the text bold where p is considered significant against a stated confidence limit.
